# Design and Challenges of Sonodynamic Therapy System for Cancer Theranostics: From Equipment to Sensitizers

**DOI:** 10.1002/advs.202002178

**Published:** 2021-03-12

**Authors:** Zhuoran Gong, Zhifei Dai

**Affiliations:** ^1^ Department of Biomedical Engineering College of Engineering Peking University Beijing 100871 China

**Keywords:** cancer theranostics, sonodynamic therapy, sonosensitizer

## Abstract

As a novel noninvasive therapeutic modality combining low‐intensity ultrasound and sonosensitizers, sonodynamic therapy (SDT) is promising for clinical translation due to its high tissue‐penetrating capability to treat deeper lesions intractable by photodynamic therapy (PDT), which suffers from the major limitation of low tissue penetration depth of light. The effectiveness and feasibility of SDT are regarded to rely on not only the development of stable and flexible SDT apparatus, but also the screening of sonosensitizers with good specificity and safety. To give an outlook of the development of SDT equipment, the key technologies are discussed according to five aspects including ultrasonic dose settings, sonosensitizer screening, tumor positioning, temperature monitoring, and reactive oxygen species (ROS) detection. In addition, some state‐of‐the‐art SDT multifunctional equipment integrating diagnosis and treatment for accurate SDT are introduced. Further, an overview of the development of sonosensitizers is provided from small molecular sensitizers to nano/microenhanced sensitizers. Several types of nanomaterial‐augmented SDT are in discussion, including porphyrin‐based nanomaterials, porphyrin‐like nanomaterials, inorganic nanomaterials, and organic–inorganic hybrid nanomaterials with different strategies to improve SDT therapeutic efficacy. There is no doubt that the rapid development and clinical translation of sonodynamic therapy will be promoted by advanced equipment, smart nanomaterial‐based sonosensitizer, and multidisciplinary collaboration.

## Graphical abstract

As a novel noninvasive cancer therapeutic modality, sonodynamic therapy (SDT) is promising to clinical translation, in spite of several problems in equipment and sensitizer. The recent development in SDT in terms of living monitoring and high accuracy treatment system is highlighted and an overview is provided for advanced sonosensitizer design. The challenge in future clinical use is also discussed.

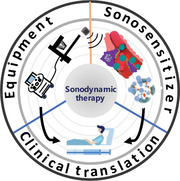

## 1 Introduction

Cancer is a high‐risk and fatal disease for humans.^[^ ^1^, ^2^ ^]^ The development of safe, advanced, and efficient cancer treatment systems is a global challenge.^[^ ^3^, ^4^, ^5^, ^6^, ^7^, ^8^, ^9^ ^]^ Though traditional chemotherapy, radiotherapy, and surgery have shown considerable effects on removing tumor tissues, they have several drawbacks such as serious side effects, low therapeutic efficacy, and immune impairment of systems.^[^ ^10^, ^11^, ^12^ ^]^ With the continuous progress in molecular imaging and molecular biology technology, cancer treatment strategies are developing toward the noninvasive and minimally invasive therapy, such as microwave coagulation therapy,^[^ ^13^, ^14^ ^]^ radiofrequency therapy,^[^ ^15^, ^16^ ^]^ phototherapy,^[^ ^17^, ^18^, ^19^, ^20^, ^21^, ^22^, ^23^ ^]^ photodynamic therapy (PDT),^[^ ^32^, ^33^, ^34^, ^35^, ^36^, ^37^, ^38^, ^39^, ^40^, ^41^, ^42^ ^]^ and sonodynamic therapy (SDT).^[^ ^24^, ^25^, ^26^, ^27^, ^28^, ^29^, ^30^, ^31^ ^]^

In PDT, a photosensitizer is administered to the patients, followed by the irradiation of the cancerous region with light to activate the photosensitizer.^[^ ^43^, ^44^ ^]^ The generated reactive oxygen species (ROS) eliminate the cancer cells. Without light, the photosensitizer is harmless. However, the clinical applications of PDT are limited by the depth of light penetration. SDT uses ultrasound (US) to activate certain sensitizers for cancer treatment. As a mechanical wave, US can penetrate much deeper tumors in human tissue than light. The different frequencies of US can be widely adopted for clinical diagnosis and treatment of cancer.^[^ ^45^, ^46^, ^47^, ^48^, ^49^, ^50^, ^51^, ^52^, ^53^ ^]^ The key effect of US is the induction of the nucleation, growth, or oscillation of bubbles on tissue, which is called the cavitation effect.^[^ ^45^ ^]^ The cavitation effect includes inertial and stable cavitation. Inertial cavitation is a process of rapid growth and bursting of bubbles. Stable cavitation is described as the process of continuous oscillation of bubbles that results in the flow rate of the liquid and applies a shear force to the surrounding tissues. When the bubbles burst, the shock wave acts in a short time and produces a large pressure gradient thus causing damage to the solid tumor.^[^ ^54^, ^55^ ^]^ Besides, cavitation has been interpreted as an improvement in drugs that are sensitive to tumor tissues, because it improves the drug delivery efficiency and reduces the dosage of chemotherapy drugs.^[^ ^49^ ^]^

However, the SDT mechanism has not been clearly illustrated. Although sufficient shear stress generated by the streaming around oscillating bubbles can cause cell destruction, known as the hydrodynamic stress, there is a challenge to attribute the cell‐damaging effects to acoustic cavitation without sonosensitizers.^[^ ^56^, ^57^, ^58^, ^59^ ^]^ The inertial cavitation can generate the reactive hydroxyl radicals and hydrogen atoms inside the sheared bubbles which react with volatile molecules due to hydrodynamic stress to generate new radicals and strengthen hydroxyl radical production. This strategy can significantly enhance the production and chemical reduction of hydroxyl radicals.^[^ ^60^ ^]^

The existence of sonoluminescence (SL) was regarded as a kind of emission light from cavitation with an unclear mechanism.^[^ ^61^ ^]^ The SL light spectrum showed a peak in the 400–450 nm region of hematoporphyrin absorption, which could activate the hematoporphyrin. He et al.^[^ ^62^ ^]^ successfully detected the SL light in vivo with low‐frequency US (40 kHz) and low acoustic pressure (0.2 MPa), demonstrating the role of SL in the sonodynamic process. The SL signals protoporphyrin IX (PpIX)‐coupled gold nanoparticles were detected under the US irradiation (1.1 MHz, 1 and 2 W cm^−2^).^[^ ^63^ ^]^ Furthermore, the cytotoxicity is caused by ROS produced by the excitation of sonosensitizers, which is similar to photodynamic therapy. Singlet oxygen was reported to play a vital role in causing cell death in the hematoporphyrin presence.^[^ ^54^, ^61^, ^63^, ^64^, ^65^, ^66^ ^]^ By analogy to that, transferring energy to oxygen for ROS has been considered as a possible mechanism for Photofrin,^[^ ^67^ ^]^ Rose bengal (RB),^[^ ^68^ ^]^ ATX‐70,^[^ ^69^ ^]^ and inflammatory drugs such as tenoxicam and piroxicam^[^ ^70^ ^]^ in the last two decades. Many scholars are now engaged in the research of sonodynamic therapy including the mechanism illustration, sensitizer development, or biomedical application.^[^ ^49^, ^50^, ^71^, ^72^, ^73^, ^74^, ^75^, ^76^, ^77^, ^78^, ^79^, ^80^, ^81^, ^82^, ^83^, ^84^, ^85^, ^86^, ^87^, ^88^, ^89^, ^90^, ^91^, ^92^ ^]^

On one hand, sonosensitizers are an indispensable component of SDT. Ideal sonosensitizers should be highly sonosensitivity, nontoxic in the absence of ultrasound, have specific accumulation at the tumor site and ability to be excreted from the body within a short period. Nonetheless, the lack of effective sonosensitizers limits the clinical applications of SDT. SDT efficacy could be improved by the use of targeted delivery strategies to deliver sonosensitizers to the tumor site specifically.^[^ ^93^, ^94^, ^95^, ^96^, ^97^, ^98^, ^99^, ^100^, ^101^, ^102^ ^]^ High drug capacity can be achieved by encapsulating conventional sonosensitizers inside nanoparticles to better the efficiency of intracellular delivery and accumulation to deep tumor sites^[^ ^103^ ^]^ due to the enhanced permeability and retention effect. Moreover, nanoparticles also provide nucleation sites to generate bubbles, thus improving the SDT effects.^[^ ^104^ ^]^ The key to guiding SDT to successful clinical translation is to develop novel nano‐sonosensitizers with good biocompatibility, high bioavailability, and specificity, superior sonodynamic efficiency, and optimization of US regulations.

On the other hand, it is crucial to develop sonodynamic therapeutic instruments adapted for clinical use. The ideal sonodynamic device needs to be committed to adjustable acoustic parameters, accurate acoustic positioning, providing a platform for sonosensitizer screening, living temperature monitoring, ROS concentration monitoring, and integration of clinical diagnostics. In practice, for a complete sonodynamic device, there is a need to concentrate on probe design, ultrasonic drive unit, ultrasonic echo signal processing, display unit, and controller unit for suitable practical use. The delicate design is especially needed to make ultrasonic imaging transducer and ultrasonic therapy transducer cooperate and minimize the interference between them. This can conduct preoperative and intraoperative guidance and realize real‐time monitoring during the treatment process.^[^ ^105^ ^]^ Therefore, it is a systematic project to construct a controllable US therapeutic system that meets clinical needs. A uniform standardization should be developed for the SDT‐involved parameters, such as power, frequency, density, and pressure of ultrasound. The solution to these problems would accelerate the SDT clinical transformation.

In this review, we outlined the major considerations for designing SDT equipment and sonosensitizers to improve SDT therapeutic efficacy by summarizing the recently developed equipment and sensitizers of SDT. We also addressed the advances and challenges of SDT systems including both equipment and sonosensitizers and provided our constructive proposals in the following discussions that aim at promoting the successful clinical translation of SDT. Moreover, the multidisciplinary collaborations would definitely promote the rapid development of SDT for cancer therapeutics.

## 2 SDT Equipment Development

### 2.1 Optimizing Acoustics Parameters Properly

Due to its in‐depth penetration into biological tissues,^[^ ^106^ ^]^ low intensity focused ultrasound (LIFU) can produce cavitation in several tissues and activate a sonosensitizer to generate ROS molecules in moderation, thereby making cytotoxicity at tumor sites without other side effects. The determination of acoustics parameter is related to the US dose, the sonosensitizer efficiency, and the lesion position. In general, the US frequency range of 150 kHz–3 MHz, irradiation dose of 2–3 W cm^−2^, and the actuation duration range of 1–20 min are used for SDT research. It was shown that the second‐harmonic superimposition could induce sonochemical effects and enhance the SDT.^[^ ^107^, ^108^, ^109^, ^110^ ^]^ A relationship between the relative phase and cavitation effect and found cavitation to be induced under a certain condition (phase interval of less than 0.5 *π*, and shift interval of more than 30 ms).^[^ ^111^ ^]^ Also, Alamolhoda and Mokhtari‐Dizaji^[^ ^112^ ^]^ found that the dual‐frequency ultrasound treatment in mouse breast cancer had the apparent SDT effect, while single‐frequency ultrasound treatment did not. Nevertheless, the use of two transducers may lower the accuracy of the ultrasound focus. Therefore, there is a pressing need to design a transducer that generates two frequencies simultaneously.^[^ ^113^ ^]^

Multi‐frequency US provides more options to optimize several parameters to improve the efficiency of SDT. Moreover, it was found that US dose fractionation could lead to higher therapeutic efficacy SDT than single irradiation.^[^ ^90^ ^]^ Compared with single irradiation, the same dose of US fractionation exhibited better suppression of tumor growth since US dose fractionation gave rise to higher ROS concentration.^[^ ^112^ ^]^ The pulsed US field (with a certain duty cycle) induces more effective cavitation than the continuous US field when US power density far exceeds the cavitation threshold.^[^ ^114^, ^115^, ^116^ ^]^ Secomski et al.^[^ ^117^ ^]^ further proved that establishing multiple reflection fields and using standard waves could achieve temperature‐controlled therapeutic modality.

### 2.2 Making Ultrasonic Irradiation Location Exactly

To ensure the therapeutic effectiveness of cancer SDT, especially for tumor sites in deep tissues, acoustic irradiation should be accurately placed. Thus, it is necessary to develop a low intensity focused ultrasonic probe that is similar to the high intensity focused ultrasound (HIFU) which performs US focusing in a concave acoustic lens or a transceiver‐based delay circuit. The comparison of the multiple phased array transducer with singlet transducer is presented in **Table** **1**.

**Table 1:** Summary of characteristics of singlet focusing inducer and multiple phased array transducers

Transducer type	Strengths	Weaknesses	Ref.
Singlet focusing	Simple structure	Prolong SDT retention time	^[^ ^118^ ^]^
Multiple phased array	Adjustment to focusing range and position exactly	Complicated ultrasonic field	^[^ ^119^, ^120^ ^]^

More to the above‐mentioned transducer arrangements, the multi‐frequency focused ultrasonic transducer should be designed to meet the needs of emitting multi‐frequency focused US in clinical use. A plurality of piezoelectric crystals is appropriately arranged in the transducer. For example, Gao ^[^ ^121^ ^]^ set multiple self‐focusing or acoustic‐focusing lenses on the same plane with the transducer at the same distance. This ensured that the multi‐frequency focused US achieved the second ultrasonic focusing instead of the complicated multi‐frequency focusing ultrasonic transducer which is made of the traditional composite piezoelectric materials.

In addition to developing low intensity focused ultrasonic probe, a variety of image guidance can provide effective support for acoustic aiming and positioning. positron emission tomography (PET)–computed tomography (CT) has developed into a reliable imaging mode that can characterize tumors based on biochemical changes at the molecular level, to locate ultrasound accurately. Indocyanine green (ICG)‐based fluorescence imaging (FI), as a widely used technique for tumor location, can also provide the exact guidance for SDT. Moreover, as shown in **Table** **2**, magnetic resonance imaging (MRI), ultrasound imaging, harmonic imaging, color Doppler imaging, and photoacoustic imaging can be used to fix the tumor location for SDT imaging guidance.

**Table 2:** Summary of image positioning used in SDT

Imaging system	Characteristics	Application	Ref.
MRI	Image with high resolution, no support to living monitoring	Fixing the tumor location	^[^ ^122^, ^123^ ^]^
PET‐CT	Image with high sensitive, based on biochemical changes in tumor cells at the molecular level	Fixing the tumor location	^[^ ^171^, ^172^ ^]^
FI	Invisible near infrared fluorescent light can provide high sensitivity, high‐resolution, and real‐time image‐guidance during oncologic surgery	Fixing the tumor location	^[^ ^126^ ^]^
USI	Support to living monitoring, avoiding the interference of US beam	Fixing the tumor location	^[^ ^124^, ^125^ ^]^
HI	Less attenuation, improving the quality of image	Fixing the tumor location	^[^ ^127^, ^128^ ^]^
CDI	Optimization and compensation of image	Fixing the tumor location	^[^ ^129^ ^]^
PAI	Living temperature monitoring and tissue statement	Fixing the tumor location	^[^ ^130^ ^]^
HMI	Detecting the maximum tissue shift point to determine the ultrasonic focal point position	Fixing the US focal point location	^[^ ^131^, ^132^ ^]^
ARFI	Acquiring the peak of shift with different axial depth for harmonic motion to determine the ultrasonic focal point position	Fixing the US focal point location	^[^ ^133^, ^134^ ^]^
SSI	Constructive interference between shear waves creates a cumulative effect that induces high‐mechanical displacements in the medium	Fixing the US focal point location	^[^ ^135^ ^]^
MR‐ARFI	Based on ARFI, short time needed for focused ultrasonic pulse signal, with low heat deposition	Fixing the US focal point location	^[^ ^136^, ^137^, ^138^ ^]^

Abbreviations: MRI: magnetic resonance imaging, FI: fluorescence imaging, USI: ultrasound imaging, HI: harmonic imaging, CDI: color Doppler imaging, PAI: photoacoustic imaging, HMI: harmonic motion imaging, ARFI: acoustic radiation force impulse imaging, SSI: supersonic shear imaging, MR‐ARFI: magnetic resonance‐ acoustic radiation force impulse imaging.

Furthermore, harmonic motion imaging,^[^ ^131^ ^]^ acoustic radiation force impulse imaging (ARFI),^[^ ^133^ ^]^ and supersonic shear imaging ^[^ ^135^ ^]^ (Table 2) are potent to fix the ultrasound focus position, for exact irradiation of the tumor. McDannold and Maier^[^ ^136^ ^]^ first introduced MRI into the acoustic radiation force imaging, demonstrating the feasibility of MR‐ARFI mode. MR‐ARFI can monitor small tissue displacement through MRI pulse sequences. Also, it has been developed in terms of imaging speed and sensitivity through several MRI pulse sequences and imaging methods.

### 2.3 Providing Living Monitoring of Treatment Parameters during the SDT

#### 2.3.1 Real‐Time Temperature Monitoring

The above‐mentioned imaging can be combined with SDT equipment to monitor the acoustics parameters. However, some key parameters, such as temperature and ROS concentration of the tumor site, should also be considered during the SDT process. For SDT, the mechanism is based on ROS or cavitation effects rather than thermal effects, and too high temperature causes irreversible thermal damage to surrounding tissues. This makes it crucial to add a temperature monitoring module to SDT devices. Many noninvasive temperature measurement methods exist, such as MRI, infrared, radiance, electrical impedance, microwave, and US. Among them, MRI can accurately obtain the cross‐sectional temperature distribution of the human body and is broadly used to carry out clinical research on the treatment of glioma cancer related to SDT.^[^ ^139^, ^140^ ^]^ To overcome the limitation of the unreal‐time monitoring of MRI, a combination of MRI with other methods has become a new point of focus in research. Dixit et al.^[^ ^141^ ^]^ attempted to monitor temperature during MRI using thermoacoustic ultrasound (TAUS), and found that the TAUS signals could accurately estimate the needle tip temperature. Bour et al.^[^ ^142^ ^]^ proposed a new acquisition sequence for multi‐slice, simultaneous, and sub‐second imaging of tissue temperature during ablation. They obtained a single‐echo planar imaging sequence to monitor local temperature rising. Based on Bour's research, Ozenne et al.^[^ ^143^ ^]^ measured the temperature changes caused by US irradiation under MRI‐guidance in unrivaled monitoring capabilities achieved by only four slices. It finally turned out that this method helped to define the safe range of operation and improved the accuracy and efficacy of treatment.

Besides, ultrasonic thermometry is an ideal choice because of its good compatibility with the SDT system. The ultrasonic thermometry obtains temperature data by establishing a functional relationship between the temperature and the parameters of certain tissues,^[^ ^144^ ^]^ such as sound velocity, attenuation characteristics, nonlinear parameters, acoustic intensity, and frequency offset of ultrasonic echo.^[^ ^145^ ^]^ Ultrasonic thermal strain imaging is a method of forming a 2D temperature distribution image by the temperature dependence of ultrasonic echo time shift and is achieved by the change of ultrasonic tissue properties in the heating area.^[^ ^124^ ^]^ By tracking the echo time shift caused by changes in sound velocity and tissue thermal expansion, the thermal strain can be calculated, and the relationship between ultrasonic velocity and temperature allows for the real‐time imaging of the temperature distribution in the heated area. To address the small error between the imaging and real temperature, it is significant to develop an effective compensation algorithm to repair the module and calculate more accurately the relationship between parameters and temperature.

Moreover, there are several new ideas on temperature monitoring. For instance, Audigier et al.^[^ ^146^ ^]^ tried to develop 2D temperature monitoring of thermal ablation based on the US brightness mode (B‐mode) imaging and thermal simulation and obtained a smaller average temperature error. Kim et al.^[^ ^147^ ^]^ designed a HIFU‐living photoacoustic thermometry system with clinical US imaging with the feasibility of achieving safe and effective monitoring during the real‐time treatment. Landa et al.^[^ ^148^ ^]^ demonstrated the optoacoustic monitored temperature variations with simultaneous thermocouple readings, and are regarded as 4D optoacoustic monitoring of tissue heating. Giurazza et al.^[^ ^149^ ^]^ conducted a preliminary analysis of thermometry based on ultrasound elastography and evaluated the feasibility of ultrasonic thermometry using specific ultrasound imaging technology based on elastography ARFI for laser ablation of biological tissue, though the results were not satisfactory. Chen et al.^[^ ^150^ ^]^ even proposed a method for monitoring temperature with ultrasonic waves using a deep learning method. Overall, the latest developments in temperature monitoring show the importance of temperature measurement in SDT.

#### 2.3.2 Real‐Time ROS Monitoring

In clinical practice, ROS is another key parameter that needs to be monitored as its concentration and distribution can reflect the effect of sonodynamic therapy in real‐time, adjust the treatment parameters timely, and optimize the treatment process. In recent years, the noninvasive monitoring of ROS has been developed and played the role of SDT. To monitor the sonodynamic in vivo, we suggested here the chemiluminescence mechanism, a phenomenon produced by several ROS chemical reactions. On this basis, various chemiluminescent probes suitable for different ROS species were developed, as shown in **Table** **3**.

**Table 3:** Summary of the analytical performance of chemiluminescence (CL) probes for ROS

Study	CL probe	Species	Luminescence wavelength	LOD	Selectivity	Sample/application	Ref.
Hu et al. (2011)	BPD‐MA	^1^O_2_	1270 nm	0.5 µg mL^−1^	Good	In vivo imaging	^[^ ^151^ ^]^
Zou et al. (2016)	TPE‐SDS	^1^O_2_	510 nm	50 × 10^−6^ m	Good	Water	^[^ ^152^ ^]^
Hananya et al. (2017)	SOCL‐CPP	^1^O_2_	515 nm	500 × 10^−6^ m	Good	Living Hela cells	^[^ ^153^ ^]^
Baumes et al. (2010)	1EP	^1^O_2_	750 nm	1 × 10^−3^ m	Good	In vivo imaging	^[^ ^154^ ^]^
Zhen et al. (2016)	SPN	H_2_O_2_	775 nm	10 mg mL^−1^	Good	In vivo imaging	^[^ ^155^ ^]^
Lee et al. (2012)	POCL NPs	H_2_O_2_	556 nm	≈0.1 × 10^−6^ m	Good	In vivo imaging	^[^ ^156^ ^]^
Chen et al. (2015)	NaHCO_3_	H_2_O_2_	634 nm	0.3 × 10^−9^ m	Good	Water	^[^ ^157^ ^]^
Liu et al. (2015)	ABEI	H_2_O_2_	440 nm	47 × 10^−15^ m	Good	Urine	^[^ ^158^ ^]^
Seo et al. (2016)	CLNP‐PPV	H_2_O_2_	690 nm	≈1 × 10^−9^ m	Good	In vivo imaging	^[^ ^159^ ^]^
Green et al. (2017)	Dioxetane	H_2_O_2_	690 nm	NP	Good	In vivo imaging	^[^ ^160^ ^]^
Zhou et al. (2016)	CdTe QD	Hydroxyl radical (·OH)	535 nm	35 × 10^−9^ m	Good	Living cells	^[^ ^161^ ^]^
Sun et al. (2016)	SiC NPs	·OH	450 nm	263.6 × 10^−9^ m	Good	PM_2.5_	^[^ ^162^ ^]^
Li et al. (2016)	PCLA	O_2_·^−^	560 nm	pm	Good	In vivo imaging	^[^ ^163^ ^]^
Bronsart et al. (2016)	Coelenterazine	O_2_·^−^	745 nm	20 × 10^−9^ m	NP	Ex vivo and in vivo imaging	^[^ ^164^ ^]^
Niu et al. (2017)	TPE‐CLA	O_2_·^−^	500 nm	0.38 × 10^−9^ m (CL)	Good	In vivo imaging	^[^ ^165^ ^]^
Lin et al. (2011)	CDs‐NaNO_2_	ONOO^−^	440 nm	53 × 10^−9^ m	Interference: ·OH	Water and milk	^[^ ^166^ ^]^
Wang et al. (2015)	Calcein@SDS‐LDH	ONOO^−^	515 nm	0.3 × 10^−6^ m	Good	Mouse plasma	^[^ ^167^ ^]^
Zhou et al. (2016)	CdTe QD	ONOO^−^	540 nm	0.1 × 10^−6^ m	Good	Living cells	^[^ ^168^ ^]^

Abbreviations: NP: not reported, BPD‐MA: benzoporphyrin derivative monoacid ring A, TPE‐SDS: tetraphenylethenesodium dodecyl sulfonate, SOCL‐CPP: singlet oxygen chemiluminescence‐cell‐penetrating peptide, 1EP: mono(endoperoxide), SPN: semiconducting polymer nanoparticles, POCL NPs: peroxalate‐based CL nanoparticles, ABEI: *N*‐(aminobutyl)‐*N*‐(ethylisoluminol), CLNP‐PPV: 2,5‐bis(diphenylamino)terephthal dicarboxyaldehyde‐*p*‐xylylene dicyanide‐bis[3,4,6‐trichloro‐2‐(pentyloxycarbonyl)phenyl] oxalate, CdTe QD: CdTe quantum dots, PCLA: CPs‐imidazopyrazinone moiety, TPE‐CLA: tetraphenylethene‐imidazopyrazinone, CDs‐NaNO_2_: carbon dots‐sodium nitrite, Calcein@SDS‐LDH: calcein‐sodium dodecyl sulfate‐layered double hydroxides.

As a word, chemiluminescence can be used to quantitatively detect ROS thereby monitoring the SDT process. It can also be a blueprint for new imaging methods for tumor location and treatment information feedback.

### 2.4 Construction of Integrated Diagnosis and Treatment System

It is of great significance to guide the combination of SDT and multiple imaging fusion. It can help integrate multiple image information and improve the in vivo imaging capacity at the tumor site.^[^ ^169^ ^]^ Multiple imaging fusions can superimpose jointly registered information on the same subject under the combination of different imaging technologies. Undoubtedly, the bimodal instruments are being called intensely in the modern imaging field. **Table** **4** summarizes recent research in the integrated imaging field.

**Table 4:** Summary of imaging fusion with ultrasound in different application

Study	Sensitizer	Ultrasound type	Imaging System	Characterization	Application	Ref.
Maria et al. (2017)	SPIONs	MMUS	PET/CT, MRI	Breaking the penetration depth limitation of MMUS	Sentinel lymph node rat model	^[^ ^170^ ^]^
Provost et al. (2018)	NP	UUS	PET/CT	Simultaneous, fully co‐registered imaging	Sdhb‐deficient tumor model in rat, CCL‐39 tumor model in rat	^[^ ^171^ ^]^
Liu et al. (2020)	^68^Ga‐PSMA	TRUS	PET/CT	Improvement of clinically significant cancer detection	Clinical prostate cancer patients	^[^ ^172^ ^]^
Wan et al. (2016)	NP	CEUS	CECT/CEMRI	Improvement of inconspicuous liver lesions on conventional ultrasound visualization	Clinical liver cancer patients	^[^ ^173^ ^]^
Yi et al. (2016)	NP	CEUS	MRI	Improvement of the detection, precise localization, and accurate diagnosis of hepatocellular carcinomas	Clinical hepatocellular carcinomas patients	^[^ ^174^ ^]^
Alford et al. (2018)	TA/PVPON	HIFU	MRI	Providing higher treatment precision	Tumor model of breast cancer in rat	^[^ ^175^ ^]^
Ma et al. (2017)	NP	B‐mode ultrasound	B‐mode ultrasound imaging	Exhibition of distinguish features of the spectrum pattern, expected to organs recognition	Ultrasound imaging on an adult mouse	^[^ ^176^ ^]^
Lee et al. (2019)	HTSC	HIFU	TSI	Visualization the 2D spatial distribution and temporal change in temperature and localize the heating region	Tumor model of breast cancer in rat	^[^ ^177^ ^]^
Guo et al. (2019)	NP	HIFU	TSI	Real time ablation thermal dose monitoring	NP	^[^ ^178^ ^]^
Kim et al. (2016)	Nanonaps	US imaging	PAI	Both PA images and US images can be acquired in real‐time	Ultrasound imaging on adult mice	^[^ ^179^ ^]^
Vilov et al. (2020)	NP	US imaging	PAI	Both PA and US imaging with super resolution	Water	^[^ ^180^ ^]^
Agrawal et al. (2020)	NP	US imaging	PAI	Using LED arrays as illumination source	In vivo vascular measurements on human finger	^[^ ^181^ ^]^
Wang et al. (2016)	NP	US imaging	PAI	Improving the resolution, but also has limitation	Tumor model of melanoma in rat	^[^ ^182^ ^]^

Abbreviation: NP: not reported, SPIONs: superparamagnetic iron oxide nanoparticles, ^68^Ga‐PSMA: 68Ga‐labeled prostate‐specific membrane antigen, TA/PVPON: tannic acid /poly(vinylpyrrolidone), HTSC: HIFU and temperature‐sensitive cerasomes, MMUS: magnetomotive ultrasound, UUS: ultrafast ultrasound, TRUS: transrectal ultrasound, CEUS: contrast‐enhanced ultrasound, PET/CT: positron emission tomography/ computed tomography, MRI: magnetic resonance imaging, CECT/CEMRI: contrast‐enhanced CT/ contrast‐enhanced MRI, TSI: thermal strain imaging, PAI: Photoacoustic imaging.

**Figure 1:**
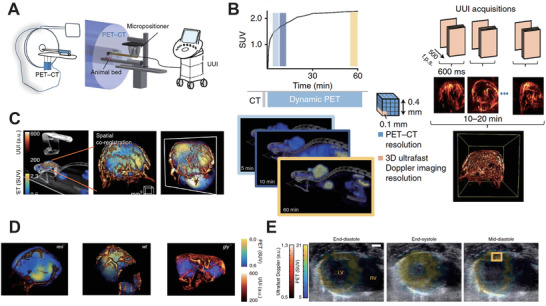
A) The schematic illustration of PET–CT–UUI trimodal imaging modality. B) Comparison of PET‐CT mode and UUI mode at the tumor site. C) Representative images of PET–CT–UUI imaging. D) Fused images of FDG uptake and perfused vessels in tumor growth. E) Representative images of metabolic activity in different timepoints. Reproduced with permission.^[^ ^171^ ^]^ Copyright 2018, Springer Nature.

In a multi‐modal medical diagnosis and treatment system, matching scan planes between different imaging modalities is a key issue to be solved. Therefore, a precious positioning sensor device should be added among several imaging systems. As shown in **Figure** **1**, Provost et al.^[^ ^171^ ^]^ designed a PET–CT–ultrafast ultrasound imaging (UUI) (PET–CT–UUI) triple‐imaging modality that integrated three imaging modes in one device. It is a system assembled with a six‐degree‐of‐freedom motorized micropositioner to obtain rigid 3D registration without marking. The effect of this marking on the volumetric mass of the ultrasound probe and PET is negligible.

The enhanced accurate SDT is based on the integration of ultrasound diagnosis and treatment process. In addition to the beneficial combination of diagnosis probes and therapeutic probes, as well as the precise coordination between the image and the treatment control system, an advanced system of high efficiency, and multifunctional sonosensitizers required for the design as a whole cannot be neglected. In the following section, we have reviewed in detail several studies in sonosensitizers development.

## 3 Sonosensitizers Design

### 3.1 Traditional Sonosensitizer Molecules

Traditional sonosensitizers are developed based on specific types of organic molecules and have been classified into four categories: porphyrins, phthalocyanines (Pcs), xanthenes, and antitumor drugs.^[^ ^183^ ^]^ Porphyrin is considered as one of the first generations of photosensitizers and has been widely used in PDT. Inspired by that, we have witnessed the rapid development of porphyrin derivatives (for example, hematoporphyrin monomethyl ether, HMME) as sonosensitizers in SDT in preliminary studies(details are shown in **Table** **5**). Phthalocyanines are the second generation of photosensitizers after porphyrin. Several studies have discussed this type of sonosensitizers, for instance, zinc phthalocyanine (ZnPcS_2_P_2_)^[^ ^184^ ^]^ triggers the generation of ROS causing subsequent apoptosis of tumor tissue under acoustic irradiation. Thus, ZnPcS_2_P_2_ can be regarded as a further potential sonosensitizer. Xanthenes including eosin, fluorescein, and rhodamine are dyes. It was shown that eosins B,^[^ ^185^, ^186^, ^187^ ^]^ and RB^[^ ^71^, ^187^, ^188^, ^189^ ^]^ triggered by ultrasound could produce effective cytotoxicity to cancer cells. RB has received considerable attention in recent years, but its further clinical application is limited due to its low efficiency in tumor accumulation capacity. To overcome this disadvantage, several kinds of RB amphiphilic derivatives have been synthesized and achieved inspired results.^[^ ^189^, ^190^, ^191^, ^192^ ^]^ Numerous antitumor drugs such as Adriamycin (doxorubicin, DOX)^[^ ^193^ ^]^ and Artemisinin^[^ ^194^ ^]^ can also be used for sonosensitizers. Furthermore, nonsteroidal antiinflammatory drugs exhibit antitumor effects under US irradiation. For instance, ciprofloxacin (CPFX) and gatifloxacin (GFLX) belong to the second‐generation fluoroquinolone antibiotics, that can cause tumor tissue apoptosis through appropriate acoustic irradiation.^[^ ^195^ ^]^ Other organic molecules including 5‐aminolevulinic acid,^[^ ^85^, ^196^ ^]^ hypocrellin B,^[^ ^197^ ^]^ and ICG^[^ ^210^ ^]^ are also active on SDT, as shown in Table 5.

**Table 5:** Summary of traditional organic sonosensitizers

Study	Sonosensitizer	Categories	Hydrophilicity	Ultrasound frequency [MHz]	Maximum intensity [W cm^−2^]	Durations [min]	Target	Application	Ref.
Yumita, et al. (1990)	Hp	Porphyrins	N	1.92	1.8	1	S180 cells	In vitro	^[^ ^61^ ^]^
Yumita, et al. (2000)	Photofrin	Porphyrins	N	1.93	6	1	S180 cells	In vitro	^[^ ^67^ ^]^
Liu, et al. (2007)	PpIX	Porphyrins	N	2.2	5	3	S180 cells	Sarcoma rat model	^[^ ^198^ ^]^
Tian et al. (2010)	HMME	Porphyrins	N	10.5	0.5	0.17	Osteosarcoma cell line UMR‐106	In vitro	^[^ ^199^ ^]^
Yumita, et al. (2010)	ATX‐70	Porphyrins	Y	1.93	6	5	HL‐60 cells	In vitro	^[^ ^200^ ^]^
Yumita, et al. (2010)	DCPH‐P‐Na(I)	Porphyrins	Y	2	6	1	S180 cells	In vitro	^[^ ^201^ ^]^
Tsuru, et al. (2012)	DEG	Porphyrins	Y	1	2	10	Human gastric cancer cell lines	Gastric cancer rat model	^[^ ^202^ ^]^
Wang, et al. (2015)	DVDMS	Porphyrins	Y	1.9	4	5–7	S180 cells	In vivo	^[^ ^203^ ^]^
Chen, et al. (2020)	DVDMS	Porphyrins	Y	0.97	3.45	3	HCT116 cells	In vivo	^[^ ^205^ ^]^
Wang, et al. (2020)	DVDMS	Porphyrins	Y	1.0	0.5	1	U‐118 MG xenograft models	In vivo	^[^ ^206^ ^]^
Chen, et al. (2011)	ZnPcS_2_P_2_	PCs	N	1	0.5	2	U251 human glioma cells	In vitro	^[^ ^184^ ^]^
Tomohiro, et al. (2016)	AlPcS_2a_	PCs	Y	3	3	1	Colon‐26 cells	Colon cancer rat model	^[^ ^207^ ^]^
Haraoka, et al. (2006)	Er	Xanthenes	Y	1.2	2.9	10	Human lymphoma U937 cells	In vitro	^[^ ^185^ ^]^
Sugita, et al.(2010)	RBD3	Xanthenes	Y	1.92	8.3	0.5	S180 cells	In vitro	^[^ ^189^ ^]^
Nonaka, M. et al. (2009)	RB	Xanthenes	Y	1	25	5	Glioma cells	C6 glioma brain rat model	^[^ ^208^ ^]^
Nomikou, et al. (2012)	RBD2	Xanthenes	Y	1	1.5	60	RIF‐1 cells	In vitro	^[^ ^192^ ^]^
S. Umemura, et al. (1997)	Adriamycin	Antitumor drugs	Y	1.93	6	1	S180 cells	In vitro	^[^ ^209^ ^]^
Solttermann, et al. (2019)	Artemisinin	Antitumor drugs	N	0.024	15	20	1,4 Dioxane	In vitro	^[^ ^194^ ^]^
Sakusabe, et al. (1999)	Piroxicam	Anti‐inflammatory drugs	N	2	1.5–3	0.5–1	S180 cells	In vitro	^[^ ^73^ ^]^
Huang, D., et al. (2004)	CPFX, GFLX, LFLX, SPFX	Anti‐inflammatory drugs	N	2	1.5–3	0.5	S180 cells	In vitro	^[^ ^195^ ^]^
Liu, B. et al. (2010)	Levofloxacin	Anti‐inflammatory drugs	Y	0.4	1	180–300	Bovine serum albumin	In vitro	^[^ ^211^ ^]^
Gao, et al. (2013)	ALA	Others	Y	1.1	2	5	Human tongue cancer SAS cell line	Tongue cancer xenograft mice model	^[^ ^196^ ^]^
Wang, et al. (2012)	HB	Others	N	1.7	0.46	0.13	HepG2 cell line	In vitro	^[^ ^197^ ^]^
Nomikou N, et al. (2012)	ICG	Others	Y	1	3.5	3	RIF‐1 cells	RIF‐1 tumor mice model	^[^ ^210^ ^]^
Komori, et al. (2009)	MB	Others	Y	2	0.24	1	S180 cells	In vitro	^[^ ^212^ ^]^
Wang, et al. (2013)	Curcumin	Others	N	0.86	2	15	THP‐1 cells	In vitro	^[^ ^213^ ^]^
Suzuki, et al. (2007)	AO	Others	Y	2	2	1	S180 cells	In vitro	^[^ ^214^ ^]^
Yumita, et al. (2013)	PHF	Others	N	2	3	15	S180 cells	Colon cancer mice model	^[^ ^215^ ^]^
Wang, et al. (2015)	Ce6	Others	N	1.90	1.6	3	4T1 cells	Breast cancer mice model	^[^ ^216^ ^]^

Abbreviation: Y: hydrophilicity, N: hydrophobicity, S180 cells: sarcoma 180 cells, Hp: hematoporphyrin, PpIX: protoporphyrin IX, HMME: hematoporphyrin monomethylether, ATX‐70: 7,12‐bis (1‐decyloxyethyl)‐Ga(III)‐3,8,13,17‐tetramethyl‐porphyrin 2,18‐dipropionyl diaspartic acid, DEG: 7,12‐bis(1‐(2‐(2‐hydroxyethoxy)ethoxy)ethyl)‐3,8,13,17‐tetramethylporphyrin‐2,18‐dipropionatomanganese, DVDMS: sinoporphyrin sodium, Er: erythrosine B, RBD (RBD2, RBD3): Rose bengal derivative (Rose bengal derivative 2, Rose bengal derivative 3), CPFX: ciprofloxacin hydrochloride, GFLX: gatifloxacin hydrate, LFLX: lomefloxacin, SPFX: sparfloxacin, ALA: 5‐aminolevulinic acid, HB: hypocrellin B, ICG: indocyanine green, MB: methylene blue, AO: acridine orange, PHF: polyhydroxy fullerenes, Ce6: Chlorin e6

**Figure 2:**
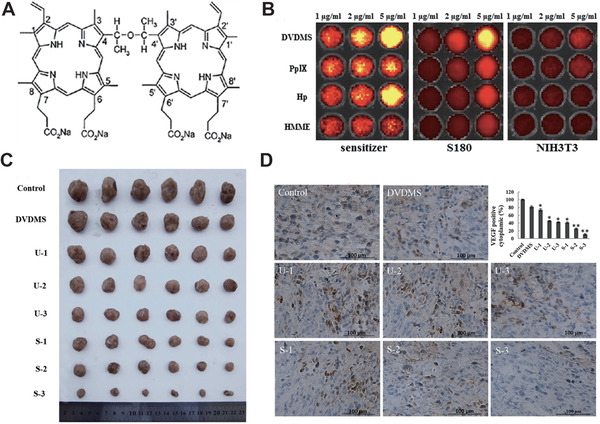
A) Chemical structure of DVDMS. B) Cellular uptake of DVDMS and other traditional sensitizers in S180 and NIH3T3 cells. C) Tumor gross morphology after 15 days of treatment. D) The VEGF expression level in tumor cells after 15 days of treatment. Reproduced with permission.^[^ ^203^ ^]^ Copyright 2015, Springer.

Because small molecules have advantages of wide use and mature theory, they are regarded as the blueprint for clinical use. As mentioned above, a series of porphyrin derivatives are potent for further SDT application, especially for the new strategies for improving SDT, sinoporphyrin sodium (DVDMS, **Figure** **2**A). Concretely, DVDMS is a kind of novel porphyrin derivatives isolated from Photofrin. Wang and co‐workers^[^ ^203^, ^204^ ^]^ undertook a series of evaluation about the effects of SDT and PDT using DVDMS on tumor inhibition both in vitro and in vivo. As a result, the accumulation of water‐soluble DVDMS in S180 (cancerous) cells is much larger than when using other sensitizers. Also, DVDMS has low accumulation in NIH3T3 (normal) cells (Figure 2B). When the S180 xenografted mice model was successfully built, the anti‐tumor efficiency of DVDMS‐mediated SDT was evaluated in vivo. It was obviously shown that ultrasound combined with DVDMS substantially inhibited tumor growth and the SDT efficiency was related to the ultrasound treatment times (Figure 2C). After DVDMS‐mediated SDT treatment, the vascular endothelial growth factor (VEGF) expression level decreased significantly (Figure 2D), indicating the inhibitory of tumor tissue development.

### 3.2 Nano/Micro‐Enhanced Sonosensitizer

**Figure 3:**
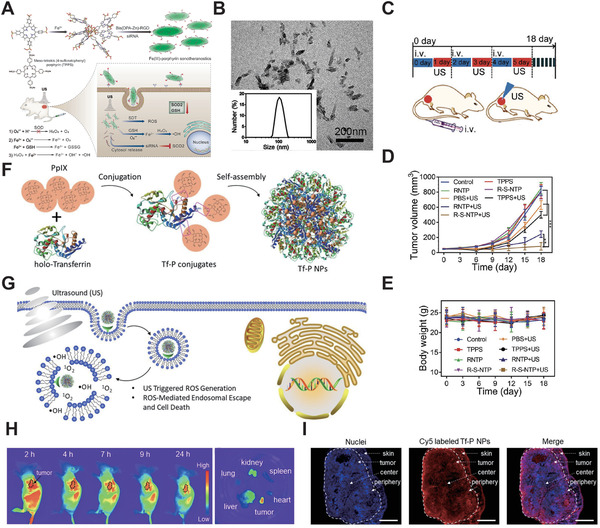
A) Schematic diagram of multifunctional sonotheranostics based on R‐S‐NTP. B) Transmission electron microscope (TEM) image of R‐S‐NTP. C) The SDT protocol in HeLa tumor‐bearing mice. D,E) Antitumor effects of SDT in several groups. Reproduced with permission.^[^ ^231^ ^]^ Copyright 2019, Wiley‐VCH. F) Schematic diagram of the synthesis of Tf‐P NPs. G) Schematic diagram of ROS‐mediate SDT therapeutics. H) Fluorescence images of Tf‐P NPs biodistribution in vivo (left) and major organs/ tumor ex vivo. I) Fluorescence images of tumor slice. Reproduced with permission.^[^ ^232^ ^]^ Copyright 2019, Wiley‐VCH.

Despite the great success of SDT based on small molecular sonosensitizers, we still suffer from their deficiencies, such as obvious nonspecificity, low stability, low bioavailability, and even serious phototoxicity.^[^ ^217^ ^]^ Recently, nanobiotechnology was applied to encapsulate and deliver small molecular sonosensitizers to improve the SDT efficacy and overcome the disadvantages of traditional SDT, opening new ways for more efficacious and safer SDT. Due to the ease of manufacturing, high biocompatibility, and satisfactory biodegradability, organic nanoparticles, such as polymer nanoparticles, liposomes, and micelles, have shown great potential clinical impacts.^[^ ^100^, ^218^, ^219^, ^220^, ^221^, ^222^, ^223^ ^]^ Moreover, the induced nanomaterials enhanced the cavitation and ROS yield to provide the augmented therapeutic efficiency.^[^ ^224^ ^]^ One of a variety of nanomaterials for synergistic therapy is metal–organic nanomaterials.^[^ ^225^, ^226^, ^227^ ^]^ The meso‐tetrakis (4‐sulfonatophenyl) porphyrin (TPPS) possesses several superior characteristics, such as hydrophilicity, biocompatibility, and high efficiency of ROS production, which need to be explored for further SDT studies.^[^ ^228^ ^]^ At the same time, there are restrictions on the clinical translation of the porphyrin derivatives due to their poor accumulation and biosafety issues.^[^ ^228^, ^229^, ^230^ ^]^ Zhu et al.^[^ ^231^ ^]^ has recently implied that the metal ions assembled in sonosensitizers could promote the sonochemical activation upon ultrasound stimuli.^[^ ^227^ ^]^ They constructed the new Fe(III)/TPPS nanostructure modified with RGD targeting molecule and siRNA (R‐S‐NTP), based on the coordinated interaction for hepatocellular carcinoma treatment. (1 MHz, 0.56 W cm^−2^, 50% duty cycle, **Figure** **3**A).

The 100 nm sized R‐S‐NTP (Figure 3B) showed good results in the evaluation of multifunctional sonotheranostics in vivo using the HepG2 tumor xenograft mouse model. After 18 days of treatment, the R‐S‐NTP group had obvious tumor growth inhibition. (Figure 3C,D) It proved the high efficiency of ROS production under R‐S‐NTP sonotheranostics treatment. No significant weight changes in mice and pathological changes in tissue was been found during the 18 days therapeutic period, indicating the relatively high therapeutic biosafety of R‐S‐NTP (Figure 3E). Overall, the “all‐in‐one” sonotheranostics nanoplatform has an advantage of its outstanding efficacy without side effects in SDT as a novel therapeutic modality, and it provides a promising paradigm for linking fundamental SDT research with clinical translations.

Zhang et al.^[^ ^232^ ^]^ prepared a new nanoplatform called Tf‐P NPs based on PpIX and iron transporting serum glycoprotein transferrin (Tf), which bind to tumor cell specifically (Figure 3F). The Tf‐P NPs could escape from endosomal and obtain efficient SDT in the case of excessive ROS generation upon US activation (Figure 3G). They evaluated Tf‐P NPs in the HeLa tumor‐bearing mice and found that Tf‐P NPs accumulate rapidly at the tumor site with a long retention time. The in‐depth penetration of Tf‐P NPs has been illustrated (Figure 3H,I) and suggest intra‐tumor formation and ROS‐mediated SDT treatment.

**Figure 4:**
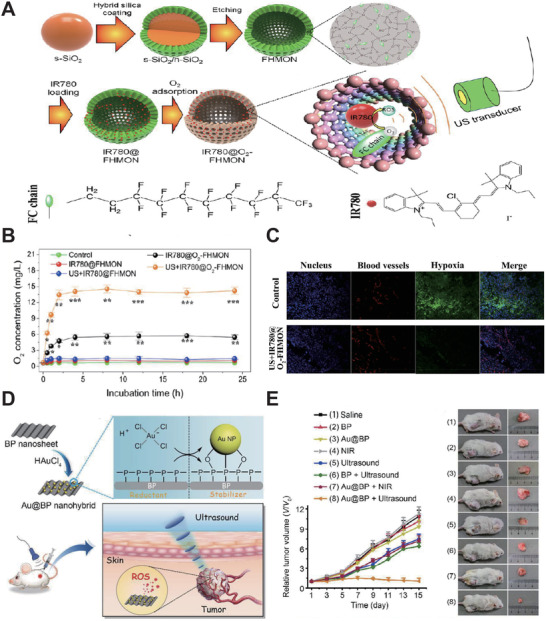
A) Schematic diagram of synthesis and enhanced SDT principle of IR780@O_2_‐FHMONs. B) Time‐sweep O_2_ concentration curves of IR780@O_2_‐FHMONs and other treatment groups in hypoxic PANC‐1 cells. C) LCSM images show the in vivo evaluation of IR780@O_2_‐FHMONs on hypoxia reversion. Reproduced with permission.^[^ ^233^ ^]^ Copyright 2017, American Chemical Society. D) Schematic diagram of Au@BP nanohybrids preparation and SDT treatment. E) Left: Tumor growth curve of Au@BP nanohybrids and other groups during 15 days treatment. Right: the antitumor effects of Au@BP. Reproduced with permission.^[^ ^234^ ^]^ Copyright 2018, The Royal Society of Chemistry.

Tumor hypoxia causes low efficiency of PDT, chemotherapy, and radiotherapy, which has plagued cancer treatment for many years. Chen et al. used IR780 as a sonosensitizer and fabricated hollow mesoporous organosilica (FHMON) based nanoplatform IR780@O_2_‐FHMONs^[^ ^233^ ^]^ to relieve tumor hypoxia and augment SDT efficiency (1 MHz, 1 W cm^−2^, **Figure** **4**A). From the perspective of FHMONs nanostructure, O_2_ could bind to a number of binding sites provided by the modified fluorocarbon (FC) chains. As shown in Figure 4B, the IR780@O_2_‐FHMON nanoplatform provides continuous oxygen generation under US irradiation, which becomes free oxygen and significantly alleviates hypoxia. Moreover, the nanoplatform displayed a considerably permanent hypoxia reversion, which benefited the efficiency of in vivo SDT (Figure 4C). Therefore, the IR780@O_2_‐FHMON based SDT was a feasible strategy for regulating hypoxia and inhibiting the growth of highly invasive hypoxic malignant tumors.

Black phosphorus (BP) as a representative semiconductor, exhibited SDT optimized with high ROS production. Ouyang et al.^[^ ^234^ ^]^ were the first group to explore the potential of BP as the new sonosensitizers for SDT (1 MHz, 1 W cm^−2^, 40% duty cycle). They fabricated BP‐based nanosheets (Au@BP nanohybrids) (Figure 4D) and found that it inhibits tumor growth in vivo effectively (Figure 4E). Therefore, the facile prepared Au@BP nanohybrids have opened a new avenue for efficient SDT and showed great potential for future study.

**Figure 5:**
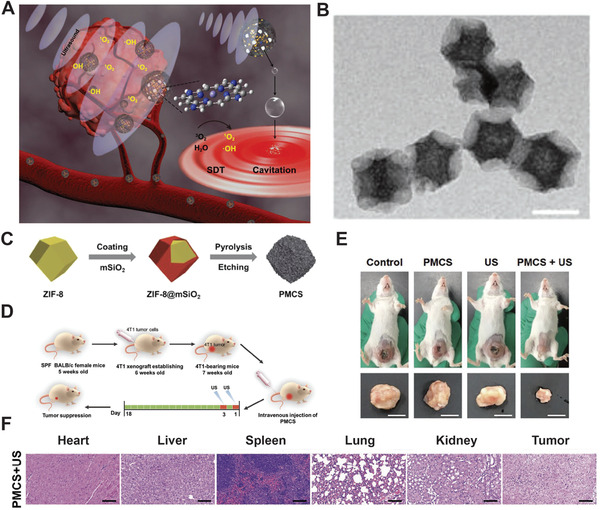
A) Schematic diagram of synthesizing PMCS. B) Representative TEM images of PMCS. Scale bar = 200 nm. C) Schematic illustration of PMCS synthesis process. D) Schematic illustration of SDT protocol in vivo. E) Treatment efficacy of PMCS. F) Biosafety evaluation of PMCS‐based SDT. Reproduced with permission.^[^ ^238^ ^]^ Copyright 2019, Wiley‐VCH.

Due to the unique properties of porphyrin derivatives such as broad‐ranging optoelectronic,^[^ ^235^ ^]^ catalytic performance,^[^ ^236^ ^]^ and their large *π*‐electron conjugated system,^[^ ^237^ ^]^ they have been intensively studied in SDT.^[^ ^235^ ^]^ Following this path, Pan et al.^[^ ^238^ ^]^ successfully synthesized a porphyrin‐like nanostructure based on metal–organic‐framework (PMCS) to achieve augmented SDT therapeutics (**Figure** **5**A,C). Their study was related to their previous work.^[^ ^239^ ^]^ The 140 nm sized PMCS (Figure 5B) (100 µg mL^−1^) could produce a certain amount of ROS under lower US intensity (1.0 MHz, 1 W cm^−2^, 50% duty cycle, 30 s). They further investigated SDT efficiency under higher US intensity (2.5 W cm^−2^) treatment periods in vivo(Figure 5D). They found that compared to the control group, the inhibition efficiency of PMCS during SDT was more than 85% with negligible damage to major organs (Figure 5E), indicating that the augmented‐SDT of PMCS could hind tumor growth without damaging the surrounding normal tissues. It is worth mentioning that the porphyrin‐like inorganic nanoparticle has high SDT efficiency with good biosafety, (Figure 5F) and has paved a new pathway for SDT with high ROS production.

**Figure 6:**
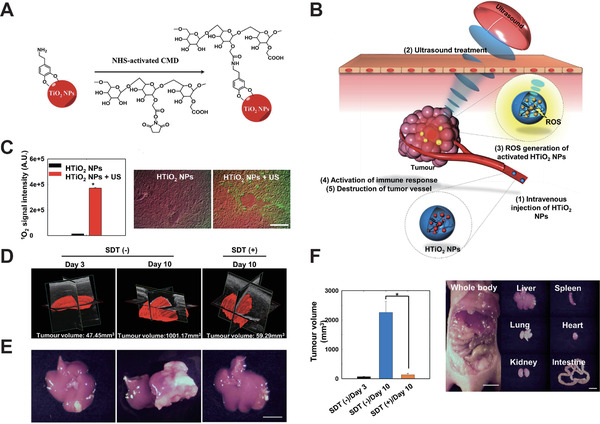
A) Surface modification of TiO_2_ NPs with carboxymethyl dextran (CMD). B) HTiO_2_ NP‐based SDT. C) Left: Quantification of ^1^O_2_ generation under the HTiO_2_ NP‐based SDT. Right: Representative ex vivo fluorescence images of ^1^O_2_ (green signal) observation induced by HTiO_2_ NP (red signal) ‐based SDT in the tumor tissue. D) Representative 3D‐rendered images of tumor volume after SDT. E) Representative images of the treated liver (Scale bar, 1 cm). F) Left: tumor volume variation after treatment. Right: representative images of major organs after SDT. Reproduced with permission.^[^ ^245^ ^]^ Copyright 2016, Springer Nature.

As aforementioned earlier, inorganic nanomaterials have been extensively studied in biomedicine owing to their multifunctionality and physiological stability.^[^ ^240^, ^241^, ^242^, ^243^, ^244^ ^]^ Many studies have focused on TiO_2_ NPs, a photosensitizer whose application was hampered by the low penetration depth of light. It has recently been demonstrated that TiO_2_ NPs could respond to the high tissue penetration of US irradiation to produce the sonocavitation effect. You et al.^[^ ^245^ ^]^ designed hydrophilized titanium dioxide nanoparticles (HTiO_2_ NPs) (**Figure** **6**A,B) and were the first group to conduct a feasibility study using HTiO_2_ NPs as an in vivo SDT sonosensitizer. They found that the presence of HTiO_2_ NPs enabled ultrasound radiation to promote the generation of ROS. Furthermore, they evaluated the ability of HTiO_2_ NPs to generate ROS in vivo during acoustics treatment (1.5 MHz, 30 W, 30 s), and found that a 29‐fold amount of ^1^O_2_ molecules existed in acoustics‐treated tumor tissue than ever before (Figure 6C). For superficial tumor tissues, the HTiO_2_ NPs successfully suppressed tumor volume and minimized damage to the vasculature during SDT treatment (Figure 6D,E). They even evaluated the effectiveness of SDT (1.5 MHz, 30 W, 30 s, 10% duty cycle) based on HTiO_2_ NPs (5 mg kg^−1^) in the deep tumor in the liver and obtained key evidence that the HTiO_2_ NP‐based SDT showed a substantial suppression of tumor growth with no sign of metastasis in the SCC7 mice liver tumor model (Figure 6F). All in all, as adjuvant therapy for deeply located tumors, HTiO_2_ NPs can effectively reduce the risk of relapse.^[^ ^246^, ^247^, ^248^ ^]^

**Figure 7:**
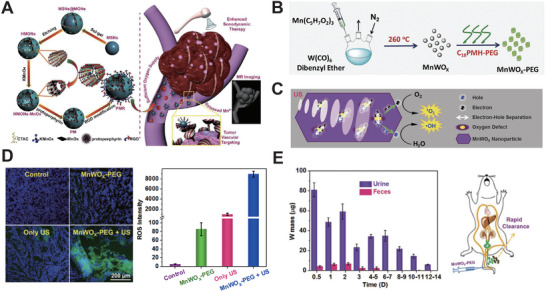
A) Schematic diagram of PMR‐based SDT modality. Reproduced with permission.^[^ ^256^ ^]^ Copyright 2018, American Chemical Society. B,C) Schematic diagram of MnWO*_x_*‐PEG‐based SDT. D) Representative image of ROS generation (left) and quantification (right) of MnWO*_x_*‐PEG. E) Left: the mass variation of W in mice body during the treatment period. Right: schematic illustration of MnWO*_x_* body clearance. Reproduced with permission.^[^ ^257^ ^]^ Copyright 2019, Wiley‐VCH.

However, both organic and inorganic sonosensitizers have several defects, which might be one of the reasons why SDT has not yet been commonly developed in clinical translation. Organic sonosensitizers show limited stability under US irradiation^[^ ^237^, ^239^, ^249^ ^]^ and may get troublesome after cancer treatment.^[^ ^250^ ^]^ Inorganic sonosensitizers, such as the TiO_2_ nanoparticle, have a low quantum yield in ROS production that limits its SDT's efficiency.^[^ ^251^ ^]^ Revisiting the SDT mechanism, we realized that the tumor microenvironment (TME) is significant to SDT efficacy. Nevertheless, the existence of glutathione (GSH) in TME exhausts ^1^O_2_ and exacerbates hypoxia in the TME. Recent studies showed manganese oxide (MnO_2_) could prevent this process effectively.^[^ ^252^, ^253^, ^254^, ^255^ ^]^ According to this mechanism and the good characteristic of HMONs, Zhu et al.^[^ ^256^ ^]^ designed a new HMONs‐based nanoplatform, named PMR, loading PpIX with RGD modified, which acted as an enzyme to catalyze the production of O_2_ under a certain US condition (1 MHz, 1.5 W cm^−2^, 50% duty cycle). The loaded PpIX generated abundant ^1^O_2_ by US irradiation (**Figure** **7**A). As the result, PMR is not only an efficient therapeutic modality but also overcomes the TME penetration limitation by US irradiation.

Gong et al.^[^ ^257^ ^]^ has synthesized oxygen‐deficient bimetallic oxide MnWO*_x_* nanoparticle, that is modified with poly(ethylene glycol) (PEG) named as MnWO*_x_*‐PEG and is classified as a combination of both organic and inorganic nanomaterials (Figure 7B). The characterization of MnWO*_x_*‐PEG showed good stability in solution and high ROS production under the triggered US (Figure 7C). When evaluating the antitumor efficacy of MnWO*_x_*‐PEG in vivo, (40 kHz, 3 W cm^−2^, 50% duty cycle, 5 min) MnWO*_x_*‐PEG activated a certain number of ROS species (Figure 7D) and caused tumor inhibition effectively. Most of W in MnWO*_x_*‐PEG would be eliminated from the body via the renal filtration pathway 14 days later, (Figure 7E) and almost metabolized after 30 days. Therefore, long‐term toxicity does not exist in MnWO*_x_*‐PEG. It is highly expected that new multifunctional nanomaterials (such as MnWO*_x_*‐PEG) will pave the way for efficient SDT.

**Figure 8:**
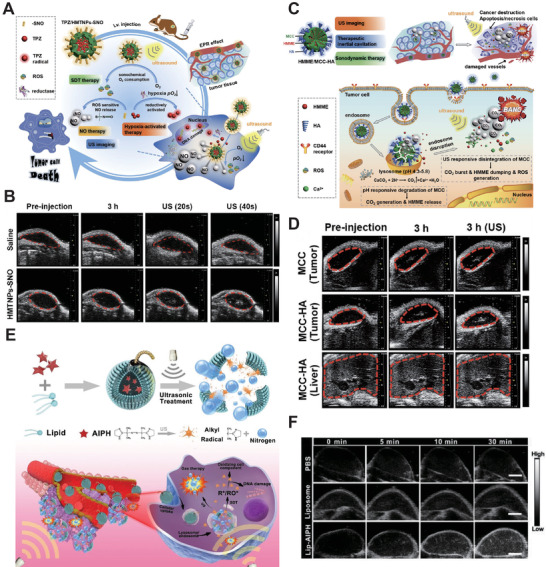
A) Schematic diagram of gas‐enhanced SDT therapy based on TPZ/HMTNPs‐SNO. B) Representative US images of TPZ/HMTNPs‐SNO. Reproduced with permission.^[^ ^258^ ^]^ Copyright 2019, Elsevier. C) Schematic diagram of HMME/MCC–HA NPs. D) Representative US images of HMME/MCC–HA in vivo. Reproduced with permission.^[^ ^259^ ^]^ Copyright 2018, Wiley‐VCH. E) Schematic illustration of Lip–AIPH liposome. F) Representative US images of tumor sites obtained post‐injection with Lip–AIPH and other groups. Reproduced with permission.^[^ ^260^ ^]^ Copyright 2019, The Royal Society of Chemistry.

Related studies have shown that gas microbubbles (MBs), which are regarded as gas therapy, can provide more nucleation and enhance the effective cavitation at the tumor site, but their uncontrolled delivery and gas release location in the body limits their application. Encountering the US with MB opens up a new method of providing timely and locally controllable gas bubbles. Thus, it is feasible to combine SDT with gas therapy to improve the therapeutic effect. Feng et al.^[^ ^258^ ^]^ constructed the ROS sensitized nitric oxide (NO) donor and formed a gas‐enhanced SDT with prepared hollow mesoporous titanium dioxide nanoparticles (TPZ/HMTNPs‐SNO) (**Figure** **8**A). TPZ/HMTNPs‐SNO exhibited a bright signal due to unlimited NO concentration under the US irradiation, (1 W cm^−2^, Figure 8B) and formed the foundation for further studies on the reversal of multidrug resistance. In another Feng et al.^[^ ^259^ ^]^ study, they constructed SDT‐carbon dioxide gas therapy based on mesoporous calcium carbonate nanoparticles loaded with HMME as sonosensitizer (HMME/MCC‐HA) (Figure 8C). For the in vivo release behavior, HMME/MCC‐HA‐based SDT exhibited uniform distribution at the tumor site, (1 MHz, 1 W cm^−2^, Figure 8D) and had significant gas‐enhanced SDT effects.

Besides, Lin et al.^[^ ^260^ ^]^ developed a synergistic therapeutic system (Figure 8E), Lip–AIPH, based on liposome loading with 2,2′‐azo‐bis[2‐(2‐imidazoline‐2‐yl)propane]dihydrochloride (AIPH) that could produce alkyl radicals in high concentration and generate N_2_ bubbles continuously under US irradiation (1.0 MHz, 2.5 W cm^−2^), which caused tumor cell apoptosis. Further, Lip‐AIPH displayed a prominent bright US image in the internal area of the tumor, (Figure 8F) indicating that Lip‐AIPH is a shine nanomaterial for gas‐enhanced SDT therapy with few side effects.

Chemotherapy is a systemic and effective modality of cancer therapeutics. However, regardless of the cause, drug resistance emerged at the tumor sites and resulted in an unfavorable prognosis among patients. Related studies showed that the US could selectively induce chemotherapeutic drugs to take up the tumor cells and reduce the side effects and toxicity to normal tissues.^[^ ^261^ ^]^ Moreover, SDT can improve the sensitivity of tumor cells toward chemotherapeutic drugs and enhance intracellular drug release. Thus, it is meaningful to form an antitumor synergy combining chemotherapy with SDT.

**Figure 9:**
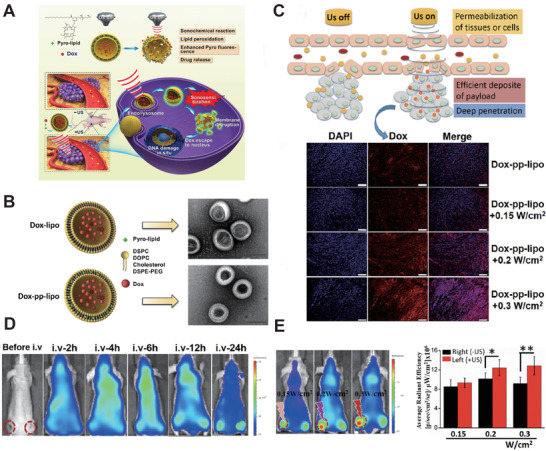
A) Schematic illustration of sonoactivatable Dox‐pp‐lipo for antitumor treatment. B) TEM image of two liposomes (Scale bar, 100 nm). C) Schematic illustration of enhanced intratumoral drug delivery and deep penetration with Dox‐pp‐Lipo under US irradiation. D) Representative image of the biodistribution of Dox‐pp‐lipo during the SDT treatment. E) Accumulation of Dox‐pp‐lipo in tumor site (left side) and radiant efficiency of Dox‐pp‐lipo (right side) under different US intensity irradiation. Reproduced with permission.^[^ ^262^ ^]^ Copyright 2018, Elsevier.

Wang et al.^[^ ^262^ ^]^ conducted a study based on the nanomaterials with high chemotherapeutic and SDT efficacy (**Figure** **9**A). In detail, they developed a DOX loading porphyrin‐based liposome (Dox‐pp‐lipo) (Figure 9B). Under the acoustics activation, the Dox‐pp‐lipo could accurately release DOX at the target location. They evaluated cellular uptake of Dox‐pp‐lipo in U87 cells under different ultrasound irradiation (Figure 9C). The results showed that the cellular uptake efficiency was improved, which promoted DOX nuclear translocation. Concretely, the enhanced penetration into tumor cell nuclei was based on the cavitation and sonochemical effects of ultrasound. Importantly, the high efficiency of LIFU (1 MHz, 0.3 W cm^−2^, 3 min) triggered drug release and antitumor in vivo. After that, the tumor‐bearing mice were irradiated with different US intensities for each different comparison group for 3 min and Dox‐pp‐lipo was used as the treatment group. Dox‐pp‐lipo exhibited statistically significant antitumor efficacy under US irradiation (Figure 9D). Interestingly, the enhanced tumor growth inhibitory effect was synchronized with increased DOX amount and US intensity. (Figure 9E) Overall, the porphyrin‐phospholipid‐liposome is expected to pave a new way for SDT clinical translation with the optimization of US parameters.

**Figure 10:**
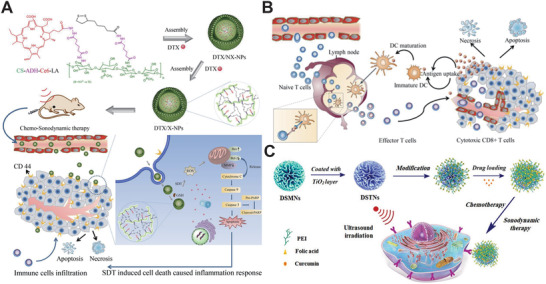
A) Schematic diagram of chemo‐sonodynamic therapy based on DTX/X NPs. B) Schematic diagram of the immune cycle induced by DTX/X NPs. Reproduced with permission.^[^ ^263^ ^]^ Copyright 2018, Elsevier. C) Schematic diagram of chemo‐sonodynamic therapy based on PEI‐FA‐DSTNs. Reproduced with permission.^[^ ^264^ ^]^ Copyright 2019, American Chemical Society.

Besides the aforementioned strategy, modifying active target molecules is a common method for effective sonosensitizer accumulation. Liu et al.^[^ ^263^ ^]^ prompted a chemo‐sonodynamic combination therapy platform for melanoma based on prepared nanoplatform loaded with docetaxel (DTX/X NPs) (**Figure** **10**A). The cluster of differentiation 44 (CD44) receptor, which is overexpressed in tumor cells, combined with the nanoplatform shell specifically as a target. A series of detailed evaluations showed that DTX/X NPs produced a large amount of ROS, leading to tumor apoptosis. Moreover, an immunological response from tumor sites was induced by tumor‐associated antigen release (Figure 10B). Generally, the DTX/X‐based chemo‐sonodynamic combination therapy platform shows a high potential to treat melanoma efficiently.

Malekmohammadi et al.^[^ ^264^ ^]^ have successfully developed a chemo‐sonodynamic system based on the dendritic mesoporous nanoparticles with silica dioxide or titanium dioxide (DSTNs) coated with polyethylenimine‐folic acid (PEI‐FA) (CUR@FA‐PEI‐DSTNs) (1 MHz, 2 W cm^−2^, Figure 10C). It had two main functions: acting as the target part enhancing DSTNs accumulation in tumor cells as well as loading CUR into the DSTNs sustainably and avoiding premature release. The results of in vitro experiments exhibited that the CUR@FA‐PEI‐DSTN system had a great responsive drug release property and obvious antitumor effects.

**Figure 11:**
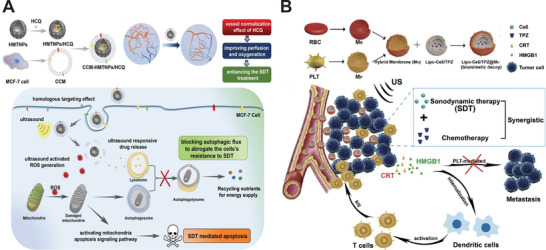
A) Schematic diagram of CCM‐HMTNPs/HCQ formulation and synergist chemo‐SDT therapy on breast cancer. Reproduced with permission.^[^ ^265^ ^]^ Copyright 2019, American Chemical Society. B) Schematic diagram of Lipo‐Ce6/TPZ@M_H_ preparation and synergistic chemo‐SDT therapy of cancer. Reproduced with permission.^[^ ^266^ ^]^ Copyright 2019, Wiley‐VCH.

Autophagy is a double‐edged sword that leads to, contributes to, aggravates, or antagonizes the tumor cell apoptosis. Even low dosage SDT can induce autophagy and may vary the direction of cancer therapeutics. Thence, it is of great significance to enhance SDT efficacy through autophagy regulation. A biomimetic system, designed by Feng et al.,^[^ ^265^ ^]^ used to regulate autophagy progress to enhance SDT efficacy. In detail, the CCM‐HMTNPs/HCQ biomimetic nanoplatform was prepared by loading the autophagy inhibitor, hydroxychloroquine sulfate (HCQ), on the aforementioned HMTNPs, modified with the cancer cell membrane (CCM) (**Figure** **11**A). CCM‐HMTNPs/HCQ showed an obvious autophagy inhibition and co‐treatment regimen of SDT (1 W cm^−2^, 30 s) on MCF‐7 cells, regarded as a synergistic therapeutic effect. When in vivo evaluation was carried, the HCQ was observed to be released into the tumor cells by US irradiation to prevent autophagic flux to eliminate the tumor cell resistance. Undoubtedly, this work allowed for a new modality for effective autophagy regulation in SDT.

Tumor hypoxia is prone to occur in sonodynamic therapy, which is an adverse reaction that seriously reduces the efficiency of SDT and significantly promotes metastasis. Zhao et al.^[^ ^266^ ^]^ tempted to solve this problem by preparing a nano biomimetic delivery system (Lipo‐Ce6/TPZ@M_H_) by loading the DNA‐breaking drug tirapazamine (TPZ) and sonosensitizer Ce6 coating with red blood cell (RBC) membrane to clear the primary and metastatic tumor effectively through chemo‐SDT synergistic therapy (Figure 11B). Owing to the RBC membrane coating, Lipo‐Ce6/TPZ@M_H_ has a long retention time via immune escape. Further, the biomimetic nanoplatform exhibited a strong tumor accumulation and almost totally eradicated the tumor and prevented tumor metastasis via SDT. Remarkably, this inspired biomimetic nano liposome to create an avenue for a promising paradigm in synergistic therapeutics for both efficient primary tumor clearance and lung metastasis suppression.

**Figure 12:**
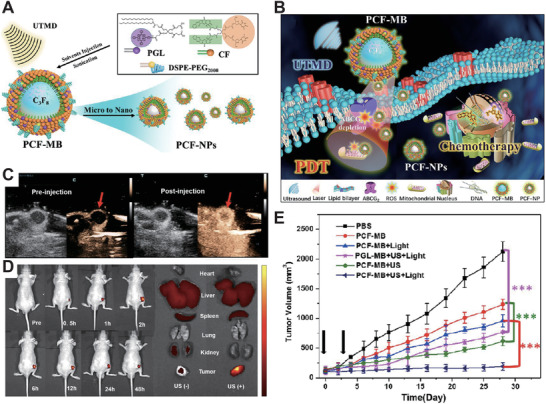
A) Schematic diagram of PCF‐MB synthesis. B) Schematic diagram of trimodal cancer therapeutics based on triad PCF‐MB. C) US images of PCF‐MB in vivo at pre‐ or post‐injection timepoint. D) Left: in vivo fluorescence images of PCF‐MB after injection at different timepoints. Right: representative images of major organs after PCF‐MB injection for 24 h. E) The trimodal treatment effects in vivo. Reproduced with permission.^[^ ^95^ ^]^ Copyright 2018, American Chemical Society.

The nanoplatform/nanocarrier‐based chemo‐SDT synergistic therapy shows higher cancer efficacy than any monotherapy, indicating the importance of co‐operative interactions between two therapies. Moreover, the multimodal synergistic therapy (such as SDT‐chemotherapy with PDT) also can enhance cancer treatment efficacy significantly. Chen et al.^[^ ^95^ ^]^ combined SDT, chemotherapy, and PDT to construct a new strategy for achieving trimodal therapy for colorectal cancer (1.0 MHz, 1 W cm^−2^, 50% duty). They first synthesized porphyrin‐based lipid (PGL) and camptothecin‐floxuridine (CF) microbubbles (PGL‐CF MBs) with high drug loading contents and stable structure with no spillage. (**Figure** **12**A,B) They evaluated the cell viability in HT‐29 cancer cells and found that PGL‐CF MBs showed enhanced cytotoxicity with increasing concentration. The PGL‐MBs significantly enhanced in vivo ultrasound imaging and extended the retention time to more than 3 min, (Figure 12C), and accumulated to the primary tumor site to a maximum extent 2 h after injection (Figure 12D). Finally, during a 30 days treatment, PGL‐MBs could almost completely inhibit tumor growth, (Figure 12E) indicating that the triad therapeutic strategy shed light on how to overcome such problems that are experienced in any monotherapy.

**Figure 13:**
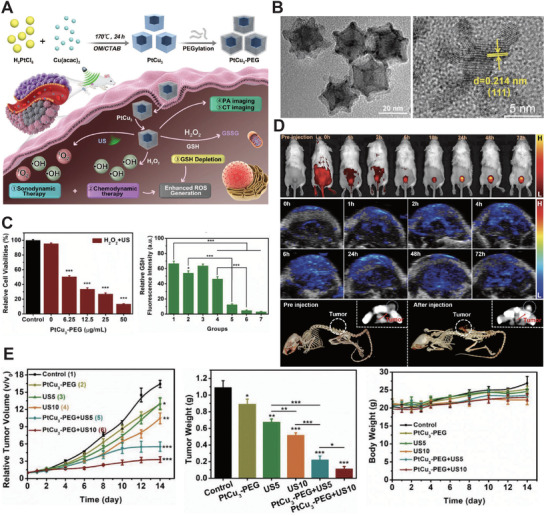
A) Schematic illustration of CDT‐enhanced SDT based on PtCu_3_‐PEG. B) Representative TEM images of PtCu_3_‐PEG nanocages. C) The effects of PtCu_3_‐PEG in vitro. D) Multimodal images of PtCu_3_‐PEG nanocages accumulation in the tumor site. E) The effects of CDT‐SDT synergistic treatment in vivo. Reproduced with permission.^[^ ^28^ ^]^ Copyright 2019, Wiley‐VCH.

Through Fenton reactions, H_2_O_2_ can be decomposed spontaneously in acidic TME, and produce toxic radical, ·OH, which is regarded as chemo‐dynamic therapy (CDT). Due to pH value differentiation between TME and normal tissues, it could only have cytotoxicity at the tumor site. Undoubtedly, it is a ROS‐consumed mechanism. SDT, as we know, can generate ROS by US irradiation in tumor sites depending on the rich oxygen environment. Thus, the CDT‐SDT synergistic therapy could relieve hypoxia to promote ROS production, enhance the tumor toxicity, and efficiently eliminate it. Yang et al.^[^ ^28^ ^]^ designed a copper‐based PEGylated nanocage (PtCu_3_‐PEG) to realize CDT‐enhanced SDT by GSH depletion (**Figure** **13**A). The TEM images showed only a 0.214 nm lattice interval and confirmed a good crystallinity (Figure 13B). Afterward, the PtCu_3_‐PEG‐based CDT‐SDT synergistic therapeutics induced a decrease in the levels of GSH in 4T1 cells, (Figure 13C) signifying that GSH was consumed by ROS produced by CDT and SDT in the deep tumor site. Multimodal images showed that PtCu_3_‐PEG accumulated efficiently at the tumor site in vivo (Figure 13D). Finally, the evaluation of CDT‐enhanced SDT of 4T1‐bearing mice indicated an exciting CDT‐enhanced SDT performance (Figure 13E). The PtCu_3_‐PEG offered a new TME‐responsive CDT‐enhanced SDT modality for deep‐seated tumors.

### 3.3 Establishment of Clinical Translation Standard for Sonosensitizer

Nowadays, we have witnessed the swift development of new sonosensitizers from the aforementioned studies on several kinds of sonosensitizers. But only a few of them have been reported to promote SDT clinical translation in cancer therapeutics. Therefore, it is necessary to focus on sonosensitizer properties and establish a standard sensitizer screening system that can promote clinical translation. Biocompatibility is the most important material characterization in clinical translation and should be evaluated preferentially. The acceptable sonosensitizers should strictly ensure that there are no toxic residues or other side‐effects on normal tissues of patients. Comparatively, the evaluation of inorganic materials is lagging behind organic materials in this respect. However, this does not mean that organic materials can be easily extended to clinical trials. In fact, the biological effects of sensitizer may change when encapsulated into nanoparticles, and most of the nanomaterials lack cytotoxicity evaluation data in vivo. Only by fully revealing the biocompatibility and biosafety of nanomaterials‐based sonosensitizers can they be introduced into further clinical trials.

On the other hand, high tumor accumulation efficiency is also crucial for the clinical translation of sonosensitizers. In other words, a promising sonosensitizer should accumulate in the targeted lesion, thereby obtaining high SDT efficacy. Typically, the oversize of a molecule objectively limits its accumulation efficiency, resulting in low tumor penetration and low SDT efficacy.^[^ ^267^ ^]^ Controlling the nanomaterial size is compulsory during the construction of nanoparticles for further improvement of SDT outcomes. As mentioned above, another factor is that the promising sonosensitizer should be sonosensitive effectively under the specific ultrasound irradiation provided by SDT equipment. In brief, the sonosensitizers should be applied to the equipment (or ultrasonic probe).

## 4 Advances and Outlook of SDT

As the frontier cancer therapeutic modality, SDT shows obvious development and is expected to make breakthroughs in clinical translation. In this part, we have summarized the advances, challenges, and outlook of each part of the SDT therapeutics modality system.

### 4.1 The Tendency of SDT Equipment Development

Nowadays, the design of SDT‐related therapeutics systems combining cancer diagnosis and treatment is a major subject for further development. Key issues such as ultrasonic positioning, SDT parameter setting, living temperature, and ROS monitoring in treated tissue are critical to the SDT efficacy in cancer therapeutics. However, the past studies have used existing ultrasonic processing instruments, or assemble experimental devices through components such as a transducer and ultrasonic probe, which may result in an unstable output of the device or inconvenient parameter adjustment. The integrated SDT system has made great progress as a result of the good compatibility between focused ultrasound and US imaging, and the ability to improve image quality constantly with little interference. Moreover, with the development of various imaging technologies, ultrasound, temperature, active oxygen concentration, and other parameters are expected to achieve real‐time monitoring and timely feedback adjustment during SDT, which will make SDT more reliable in further clinical application. Additionally, with the progress in the internet and artificial intelligent instrument technology, the combination of big databases and the addition of intelligent management systems^[^ ^268^ ^]^ will push forward the development of SDT equipment to effectiveness, security, and intelligence. Undoubtedly, these new technologies will strongly promote SDT as a promising frontier cancer therapeutics modality for further clinical translation.

### 4.2 Clinical Translations of Sonosensitizers

Sonosensitizers play an important role in SDT. Therefore, clinical translations of sonosensitizers are determined to take SDT into clinical cancer therapeutics. As we mentioned above, biocompatibility and biodegradability of sonosensitizer are the priorities of screening. Not only should we fully evaluate the side effects that cause cytotoxicity or insufficient biosafety, but also focus on the sonosensitizers with tumor accumulation effects, that is, the design and manufacture of small‐sized nanomaterials to improve the SDT outcome.

### 4.3 Feasibility of Clinical Application for SDT Modality

SDT can be broadly used as it can overcome the deficiencies of PDT. One potential direction is SDT‐based synergistic therapy, which indicates the enhancement of cancer treatment combined with chemotherapy. As Achilefu and co‐workers^[^ ^269^ ^]^ designed a photosensitizer combining radiotherapy and PDT, it is expected that a similar molecule to act as both sonosensitizer and radiosensitizer will be constructed to facilitate clinical trials of SDT. Moreover, SDT has the potential to be combined with gene therapy for gene‐transfection enhancement.^[^ ^270^ ^]^ Based on these cases, it is confident to point out that there is still more space to explore more synergistic therapeutics research to promote cancer treatment.

### 4.4 Deepen Multidisciplinary Collaboration

The research of SDT involves multidisciplinary fields such as biology, medicine, ultrasound physics, chemistry, material science, electronics, and industrial design. Strengthening the collaboration among researchers on these subjects can definitely promote the swift development of SDT cancer therapeutics.

By looking back at traditional chemotherapy or radiotherapy, we can realize that the noninvasive SDT is featured with much superiority, including low side effects, lack of radiation, and good patient compliance, showing great prospects in the clinical translations of cancer therapeutics. It should be noted that several critical issues need to be solved urgently in SDT‐based cancer therapeutics. The use of nanomaterials shows the capability to overcome the penetration depth limitation and has become the fast‐developing and promising prospective sensitizers for clinical translation. Due to its initial high performance, SDT attracts many scientific communities to devote much attention to reveal its mechanism and promote the development of nano‐based sonosensitizers. Furthermore, due to the multidisciplinary research of SDT, its rapid development will also promote the development of related disciplines such as biomedicine, ultrasound physics, material science, electronics, and industrial design. We believe that with the joint development of multiple disciplines, the sonodynamic therapy system will eventually be applied to cancer theranostics.

## Conflict of Interest

The authors declare no conflict of interest.
